# Lamin A/C augments Th1 differentiation and response against vaccinia virus and *Leishmania major*

**DOI:** 10.1038/s41419-017-0007-6

**Published:** 2018-01-08

**Authors:** Raquel Toribio-Fernández, Virginia Zorita, Vera Rocha-Perugini, Salvador Iborra, Gloria Martínez del Hoyo, Raphael Chevre, Beatriz Dorado, David Sancho, Francisco Sanchez-Madrid, Vicente Andrés, Jose-Maria Gonzalez-Granado

**Affiliations:** 1 Centro Nacional de Investigaciones Cardiovasculares Carlos III (CNIC), Madrid, Spain; 2 Servicio de Inmunología, Hospital de la Princesa, Instituto de Investigación Sanitaria La Princesa (IIS Princesa), Madrid, Spain; 3 CIBER de Enfermedades Cardiovasculares, Madrid, Spain; 4 Instituto de Investigación Sanitaria Hospital 12 de Octubre (imas12), Madrid, Spain

## Abstract

Differentiation of naive CD4^+^ T-cells into functionally distinct T helper (Th) subsets is critical to immunity against pathogen infection. Little is known about the role of signals emanating from the nuclear envelope for T-cell differentiation. The nuclear envelope protein lamin A/C is induced in naive CD4^+^ T-cells upon antigen recognition and acts as a link between the nucleus and the plasma membrane during T-cell activation. Here we demonstrate that the absence of lamin A/C in naive T-cell reduces Th1 differentiation without affecting Th2 differentiation in vitro and in vivo. Moreover, *Rag1*^*−/−*^ mice reconstituted with *Lmna*^*−/−*^CD4^+^CD25^*−*^ T-cells and infected with vaccinia virus show weaker Th1 responses and viral removal than mice reconstituted with wild-type T-cells. Th1 responses and pathogen clearance upon *Leishmania major* infection were similarly diminished in mice lacking lamin A/C in the complete immune system or selectively in T-cells. Lamin A/C mediates Th1 polarization by a mechanism involving T-bet and IFNγ production. Our results reveal a novel role for lamin A/C as key regulator of Th1 differentiation in response to viral and intracellular parasite infections.

## Introduction

The nuclear envelope is composed of nuclear pore complexes, the outer and inner nuclear membranes, and the nuclear lamina. The nuclear lamina is a filamentous protein layer mainly composed of A- and B-type lamins and provide mechanical stability to the inner nuclear membrane, regulating nucleus positioning, chromatin structure, nuclear pore complex organization, nuclear envelope breakdown and reassembly during mitosis, DNA replication, DNA damage responses, cell-cycle progression, cell differentiation, cell polarization during cell migration, and transcription^1,2^. We have previously shown that lamin A expression is triggered in naive T-cells upon antigen recognition and enhances T-cell activation by coupling actin dynamics and immunological synapse formation^3^.

T-cells orchestrate the protection against microbial pathogens^4^. In peripheral lymphoid organs, antigen-presenting cells (APCs) stimulate cognate CD4^+^ T-cells, which proliferate and undergo differentiation into distinct specialized effector T helper (Th) cells that are essential for the development of adaptive immune responses^5^. Tight control of naive T-cell differentiation is crucial for eliciting an appropriate host defense, triggering immune-mediated inflammation without deleterious tissue damage. Th subsets are defined by the differential expression of surface markers, transcription factors, and effector cytokines and play essential and distinct roles in mediating or directing the nature of the response to pathogens, commensals, and vaccines. T-cell differentiation in diverse Th subsets depends on the type of antigen encountered, the TCR signal intensity, and the local cytokine milieu^4,6–8^. Indeed, Th1 differentiation, which is required for host defense against intracellular pathogens, involves interferon-γ (IFNγ) production in an interleukin (IL)-2-dependent manner by the transcription factor T-bet^6^. Th2 differentiation is triggered by extracellular pathogens or allergens through the induction of GATA-3 and the activation of IL-4-dependent signal transducer and activator of transcription factor 6 (Stat-6)^9^. Signals emanating from the nuclear interior may also condition naive T-cell polarization. Here we show that lamin A/C expression augments CD4^+^ T-cell Th1 differentiation in response to pathogen infection by regulating T-bet transcription factor expression and IFNγ production.

## Results

### Lamin A/C regulates Th1 differentiation

**Fig. 1:**
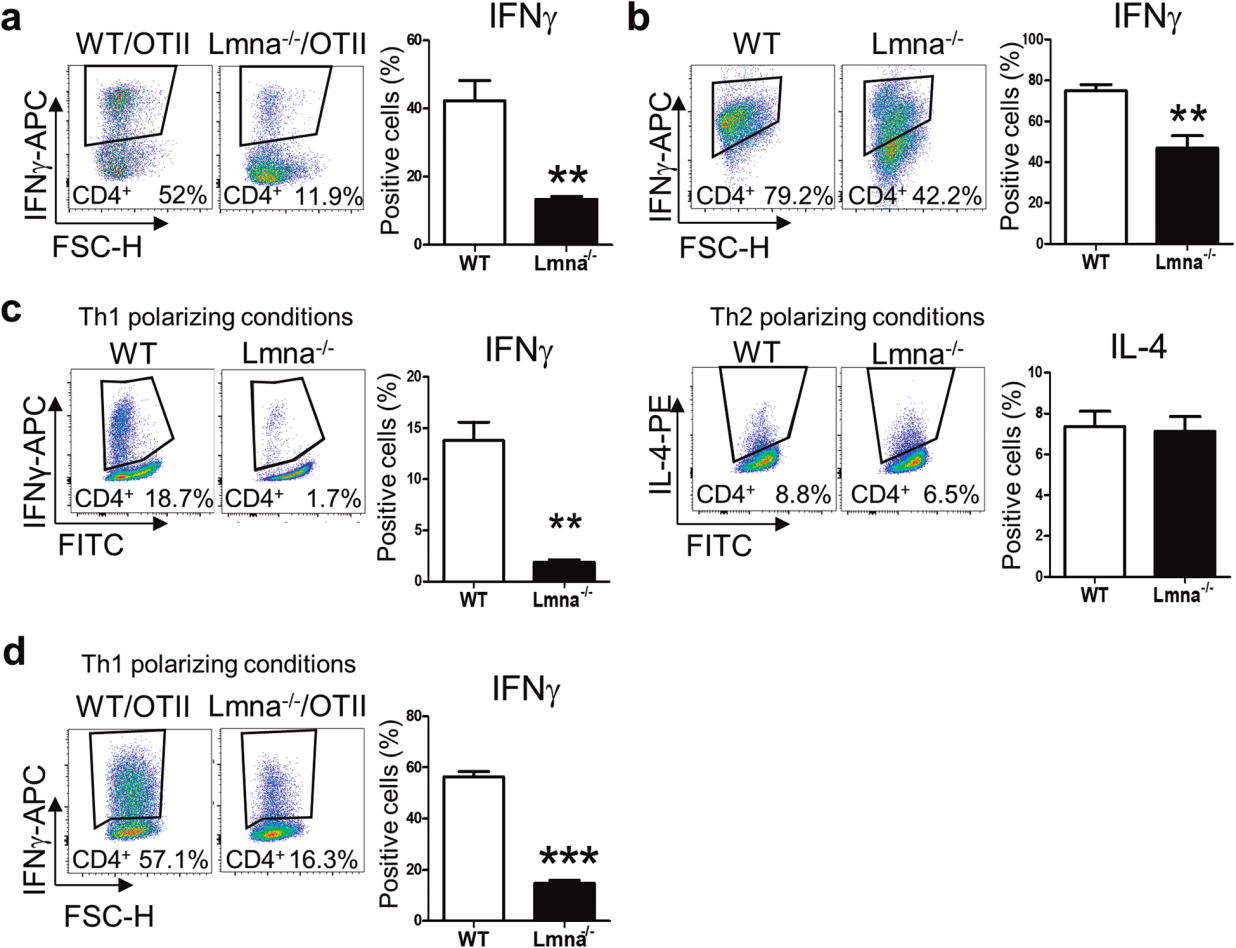
Impaired in vitro Th1 differentiation of CD4^+^ T-cells from *Lmna*^−/−^ mice Percentage of IFNγ^+^ and IL4^+^ CD4^+^ T-cells isolated from WT or *Lmna*^*−/−*^ mice. **a** CD4^+^ T-cells from WT/OTII or *Lmna*^*−/−*^/OTII mice were cocultured with OVA-loaded WT APCs for 4 days. Data are means±SEM of at least three independent experiments analyzed by unpaired Student’s *t*-test. **b** CD4^+^ T-cells were stimulated with anti-CD3/CD28 antibodies for 4 days in the presence of IL-2. Data are means±SEM of at least five independent experiments analyzed by unpaired Student’s *t*-test. **c** CD4^+^ T-cells were stimulated with anti-CD3/CD28 antibodies for 4 days in the presence of cytokines and antibodies to trigger Th1 or Th2 differentiation in vitro. Data are means±SEM of at least four independent experiments analyzed by unpaired Student’s *t*-test. **d** CD4^+^ T-cells from WT/OTII or *Lmna*^*−/−*^/OTII mice were cocultured with OVA-loaded WT APCs for 4 days in the presence of cytokines and antibodies to trigger Th1 differentiation. Data are means±SEM of three independent experiments analyzed by unpaired Student’s *t*-test. **P < 0.01; ***P < 0.001

To analyze the role of A-type lamins in antigen-dependent T-cell differentiation, *Lmna*^*−/−*^ and wild-type (WT) mice were back-crossed with OTII mice, which express a TCR (T-cell receptor) specific for ovalbumin (OVA) peptide. Naive CD4^+^ T-cells were isolated from *Lmna*^*−/−*^*/*OTII or WT/OTII mice and co-cultured with OVA-loaded WT APCs in the absence of polarizing cytokines. Compared with WT CD4^+^ T-cells, fewer *Lmna*^*−/−*^ CD4^+^ T-cells were IFNγ^+^, indicating the importance of lamin A/C for antigen-dependent Th1 differentiation (Fig. 1a). This difference was not abolished by addition of IL-2 (Fig. 1b). We next directed Th1 or Th2 differentiation in vitro by incubating WT and *Lmna*^*−/−*^ CD4^+^ T-cells with anti-CD3 and anti-CD28 antibodies and Th1 or Th2 polarizing cytokines. Interestingly, *Lmna*^*−/−*^ CD4^+^ T-cells produced fewer Th1 cells than WT cells but similar numbers of Th2 cells (Fig. 1c). Th1 differentiation triggered by co-culture with OVA-loaded WT APCs in the presence of Th1 polarizing cytokines was also lower in CD4^+^ T-cells from *Lmna*^*−/−*^*/*OTII mice than in WT/OTII T-cells (Fig. 1d). These experiments suggest that lamin A/C is an important intrinsic regulator of T-cell differentiation following TCR stimulation.

### Lamin A/C enhances Th1 responses in vivo

It has been previously shown that intrinsic lamin A/C does not affect T-cell development^10^. Moreover, we did not observe any role for lamin A/C in very early T-cell activation. Indeed, TCR stimulation triggered similar levels of extracellular signal–regulated kinase 1/2 (ERK1/2) phosphorylation in WT and *Lmna*^*−/−*^ CD4 T-cells (Figure S1a, day 0), indicating that lamin A/C is not involved in T-cell development and early TCR activation. We have previously shown that lamin A/C is transiently expressed in CD4^+^ T-cells upon antigen recognition^3^. Confirming our previous observation, levels of pERK1/2 were increased in WT lamin A/C-expressing cells but not in *Lmna*^*−/−*^ CD4 T-cells after a second TCR stimulation, when lamin A/C is already expressed in WT CD4 T-cells (ref. 3; and Figure S1b), (Figure S1a, day 1).

**Fig. 2:**
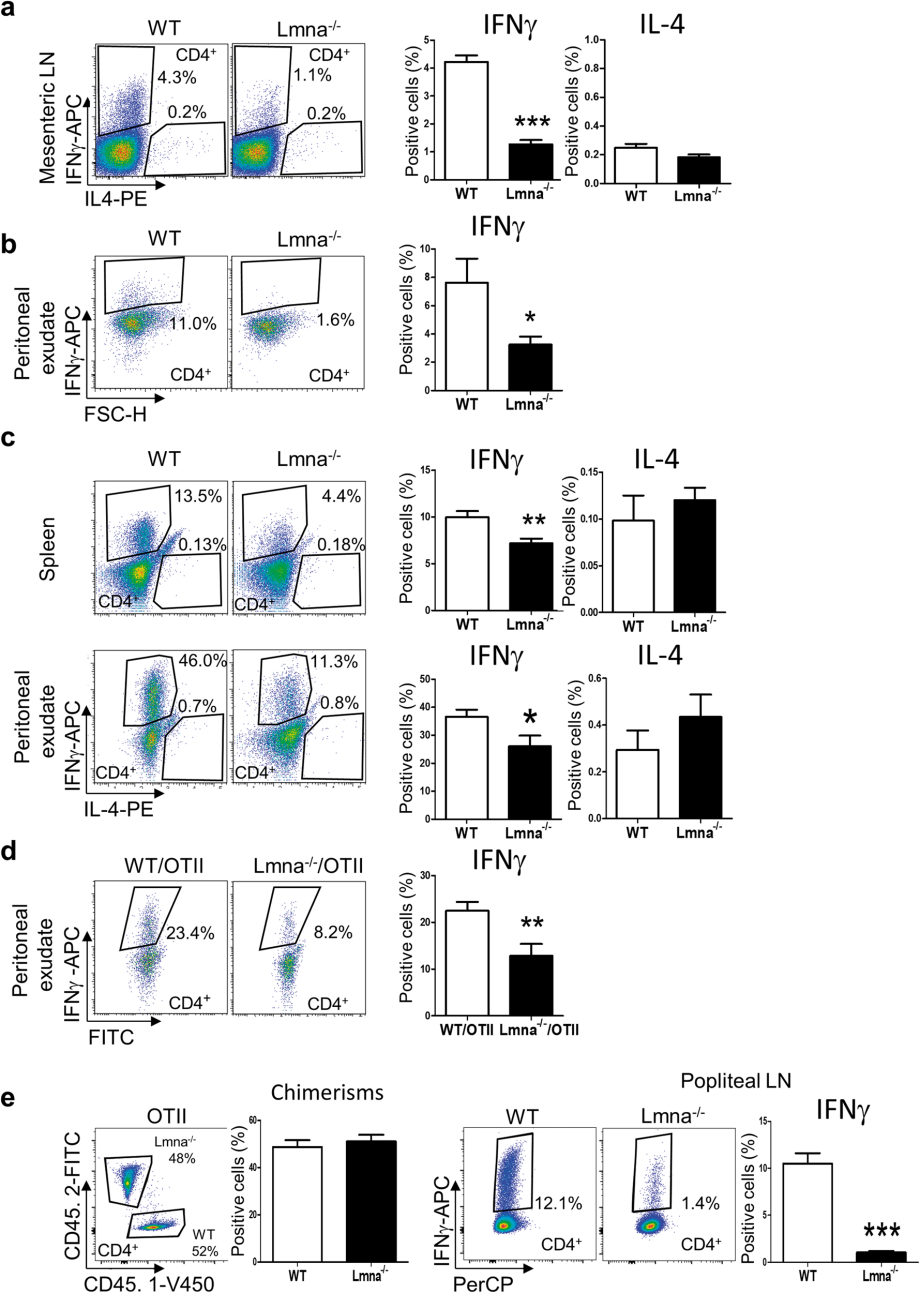
Impaired in vivo Th1 differentiation of naive CD4^+^ T-cells from *Lmna*^−/−^ mice Percentage of IFNγ^+^ or IL4^+^ CD4^+^ T-cells upon VACV infection. **a**,** b** WT and *Lmna*^*−/−*^ mice were intraperitoneally infected with VACV, and after 3 days mesenteric lymph nodes **a** and peritoneal exudate **b** were analyzed, (*n* = 11 WT and 7 *Lmna*^*−/−*^ mice). Data are means±SEM analyzed by unpaired Student’s *t*-test. **c** Irradiated CD45.1^+^ WT mice were reconstituted with CD45.2^+^ WT or CD45.2^+^ *Lmna*^*−/−*^ bone marrow and infected intraperitoneally with VACV. After 5 days, spleens and the peritoneal exudate were analyzed by flow cytometry (*n* = 7 and 8–11 mice from two independent experiments). Data are means±SEM analyzed by unpaired Student’s *t*-test **d** Irradiated CD45.1^+^ WT mice were reconstituted with CD45.2^+^ WT/OTII or CD45.2^+^ *Lmna*^*−/−*^/OTII bone marrow and infected intraperitoneally with VACV-OVA. After 5 days, peritoneal exudate was analyzed (*n* = 9 and 6 mice from 2 independent experiments). Data are means±SEM analyzed by unpaired Student’s *t*-test. **e** Irradiated CD45.1^+^/CD45.2^+^ WT mice were reconstituted with a mix of CD45.1^+^ WT/OTII with CD45.2^+^ *Lmna*^*−/−*^/OTII bone marrow. Five days after VACV-OVA intradermal infection in the footpad, popliteal lymph nodes were analyzed (*n* = 9 and 9 mice from 2 independent experiments). Data are means±SEM analyzed by paired Student’s *t*-test. *P < 0.05; **P < 0.01; ***P < 0.001

**Fig. 3:**
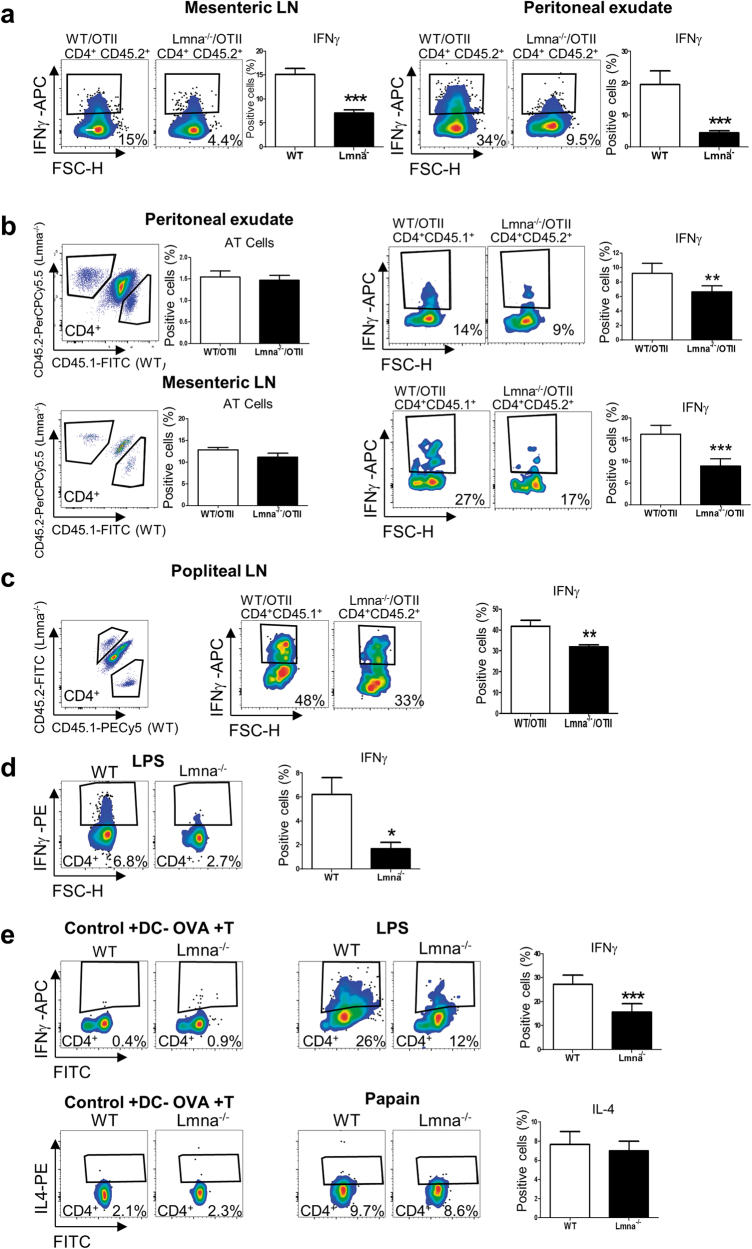
Compromised in vivo Th1 differentiation of naive CD4^+^ T-cells from *Lmna*^−/−^ mice Percentage of IFNγ^+^ CD4^+^ T-cells. **a** Recipient CD45.1^+^ WT mice were adoptively transferred with CD45.2^+^ WT/OTII or CD45.2^+^ *Lmna*^*−/−*^/OTII CD4^+^ T-cells. Five days after intraperitoneal VACV-OVA infection, mesenteric lymph nodes and the peritoneal exudate were analyzed (*n* = 10 WT and 9 *Lmna*^*−/−*^ mice from 2 independent experiments). Data are means±SEM analyzed by unpaired Student’s *t*-test. **b** CD45.1^+^/CD45.2^+^ WT mice were adoptively transferred (AT) with a mix of CD45.1^+^ WT/OTII and CD45.2^+^ *Lmna*^*−/−*^/OTII CD4^+^ T-cells and infected intraperitoneally with VACV-OVA. After 5 days, peritoneal exudate and mesenteric lymph nodes were analyzed (*n* = 11 and 11 mice from 2 independent experiments) Data are means±SEM analyzed by paired Student’s *t*-test. **c** CD45.1^+^/CD45.2^+^ WT mice were AT as in **b**, and after 5 days of intradermal infection with VACV-OVA the popliteal lymph nodes were analyzed (*n* = 6 and 6 mice from 2 independent experiments). Data are means±SEM analyzed by paired Student’s *t*-test **d** OVA-loaded LPS-matured BMDCs were AT to WT recipient mice. After 18 h, recipients received intravenous injections of a mix of CD45.1^+^ WT and CD45.2^+^ *Lmna*^*−/−*^/*OTII* CD4^+^ naive T-cells. Four days later, the popliteal lymph nodes were analyzed (*n* = 3 and 3 mice from 1 representative experiment out of 2). Data are means±SEM analyzed by unpaired Student’s *t*-test **e** Isolated splenic CD11c^+^ cells were incubated with OVA and LPS or with OVA and papain for 4 h and then AT to WT recipients. Control mice were transferred with CD11c^+^ cells in the absence of OVA (DC-OVA). After 18 h, recipients received intravenous injections of a mix of CD45.1^+^ WT and CD45.2^+^ *Lmna*^*−/−*^ CD4^+^ naive T-cells. Four days later, the popliteal lymph nodes were analyzed. The percentage of IL4^+^ CD4^+^ T-cells was also investigated (*n* = 4 and 4 mice from 2 independent experiments). Data are means±SEM analyzed by unpaired Student’s *t*-test. *P < 0.05; **P < 0.01; ***P < 0.001

To investigate the role of lamin A/C in Th1 differentiation in vivo, mice were infected with vaccinia virus (VACV), which provokes a robust Th1 immune response in mice^11,12^. VACV infection induces transient expression of lamin A/C, peaking at 1 day after infection in draining lymph nodes (Figure S2). At 3 days after intraperitoneal VACV infection, the frequency of IFNγ^+^CD4^+^ T-cells in mesenteric lymph nodes and peritoneal exudate was lower in *Lmna*^*−/−*^ mice than in WT mice (Fig. 2a, b). To study the role of lamin A/C specifically in the immune system, we reconstituted lethally irradiated WT CD45.1 mice with WT or *Lmna*^*−/−*^ CD45.2 bone marrow for 2 months. Confirmation of bone marrow reconstitution with anti CD45.1 and CD45.2 antibodies revealed no significant differences between genotypes. After intraperitoneal infection with VACV, we analyzed the frequency of IFNγ^+^ and IL-4^+^ in CD4^+^ T-cells. *Lmna*^*−/−*^-reconstituted mice had proportionately fewer IFNγ^+^ T-cells, and similar numbers of IL4^+^ T-cells with respect to WT-reconstituted mice, in both spleen and peritoneal exudate (Fig. 2c). To trigger antigen-specific Th1 responses in vivo, we reconstituted WT mice with either WT/OTII or *Lmna*^*−/−*^/OTII bone marrow and then infected the mice intraperitoneally with VACV-OVA. As before, the *Lmna*^*−/−*^/OTII-reconstituted CD4^+^ population included a lower proportion of IFNγ^+^ cells than did WT/OTII-derived CD4^+^ cells (Fig. 2d). Moreover, reconstitution with a mix of bone marrow from WT/OTII and *Lmna*^*−/−*^/OTII mice produced a lower percentage of IFNγ^+^CD4^+^ T-cells in *Lmna*^*−/−*^/OTII than in WT/OTII cells (Fig. 2e). Our results indicate that lamin A/C depletion in the hematopoietic compartment impairs Th1 differentiation in vitro and in vivo. To directly assess the importance for Th1 differentiation of lamin A/C in CD4^+^ T-cells, we adoptively transferred WT mice with CD4^+^ T-cells from WT/OTII or *Lmna*^*−/−*^/OTII mice. Th1 differentiation from *Lmna*^*−/−*^ CD4^+^ T-cells was weaker than from their WT counterparts, producing a lower percentage of IFNγ^+^CD4^+^ T-cells (Fig. 3a). Similar results were obtained when WT recipient mice were transferred with a mix of WT/OTII and *Lmna*^*−/−*^/OTII CD4^+^ T-cells followed by VACV-OVA inoculation, either intraperitoneally (Fig. 3b) or subcutaneously in the footpad (Fig. 3c). These results indicate that lamin A/C expression specifically regulates CD4^+^ T-cell differentiation toward Th1 cells in vivo.

We next investigated whether the action of lamin A/C in CD4^+^ T-cell polarization was related to antigen presentation by dendritic cells (DC). WT mice received subcutaneous injections of lipopolysaccharide (LPS)-matured OVA-loaded bone marrow-derived DCs (BMDCs), which trigger Th1 differentiation^13^, and 18 h later received intravenous injections of WT/OTII or *Lmna*^*−/−*^/OTII CD4^+^ T-cells. After 7 days, Th1 differentiation of adoptively transferred T-cells was analyzed in draining lymph nodes. *Lmna*^*−/−*^/OTII CD4^+^ T-cells produced fewer IFNγ^+^CD4^+^ T-cells in the presence of LPS-treated BMDCs than WT/OTII cells (Fig. 3d). In other experiments, WT mice received subcutaneous injections of splenic CD11c^+^ DCs incubated 4 h in medium containing LPS or papain to potentiate antigen-dependent T-cell differentiation to Th1 or Th2, respectively^13,14^. As before, 18 h later the mice received intravenous injections of WT/OTII or *Lmna*^*−/−*^**/**OTII CD4^+^ T-cells, and Th1 and Th2 differentiation was analyzed in draining lymph nodes after 7 days. *Lmna*^*−/−*^ CD4^+^ T-cells produced fewer IFNγ^+^CD4^+^ T-cells in the presence of LPS-treated BMDCs than their WT counterparts; in contrast, in the presence of papain-treated BMDCs the proportion of IL4^+^ cells was similar in WT and *Lmna*^*−/−*^ CD4^+^ T-cells (Fig. 3e). These results indicate that lamin A/C expression specifically regulates CD4^+^ T-cell differentiation toward Th1 cells in vivo.

### Lamin A/C enhances T-cell activation but is not essential for T-cell proliferation

**Fig. 4:**
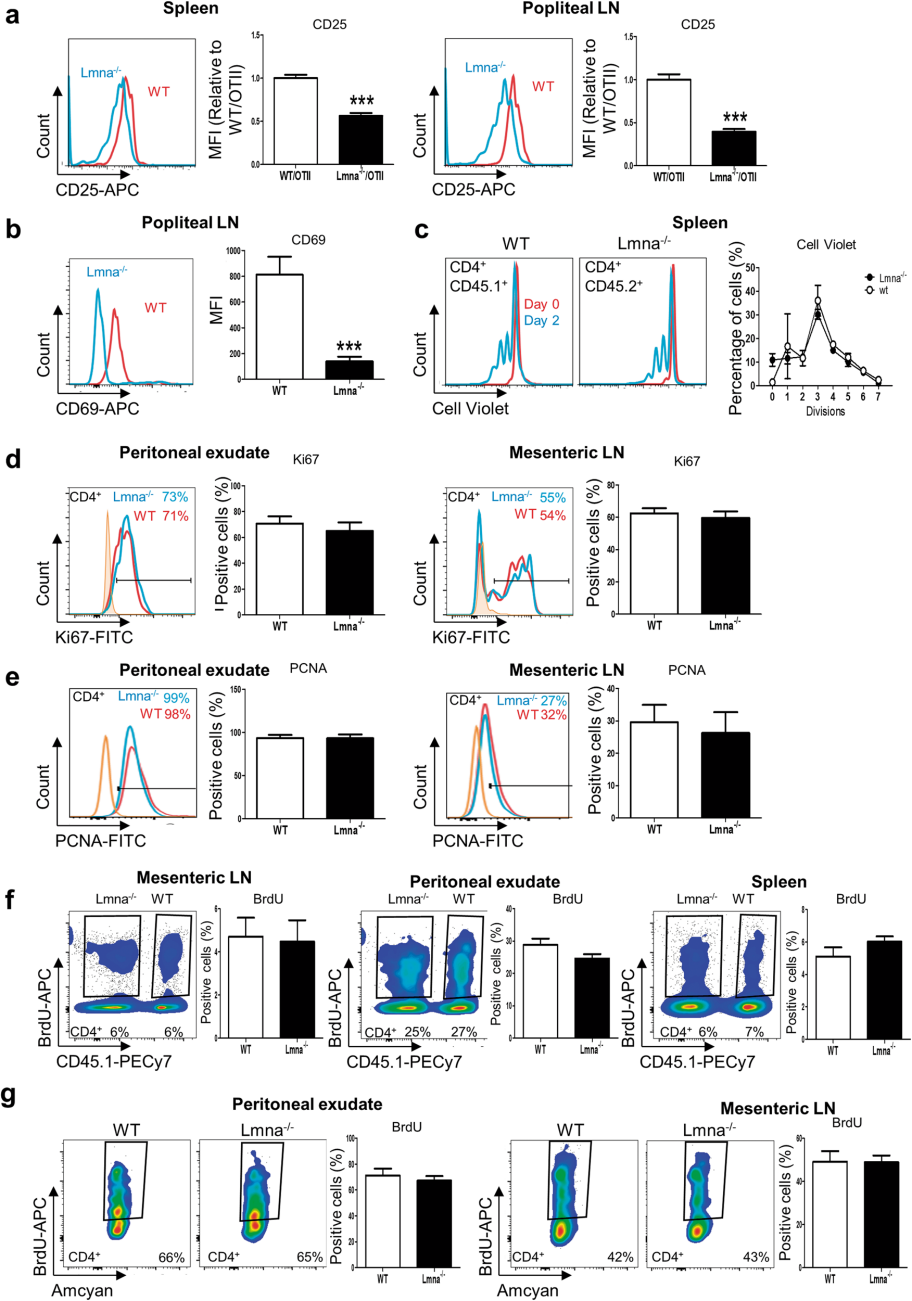
*Lmna*^*−/−*^ CD4^+^ T-cells show impaired in vivo and in vitro activation in response to VACV infection **a**, **b** WT CD45.1^+^/CD45.2^+^ mice were adoptively transferred with a mix of CD4^+^ T-cells from CD45.1^+^/OTII WT and CD45.2^+^/OTII *Lmna*^*−/−*^ mice and then intradermally infected in the footpad with VACV-OVA for 2 days. Spleen and popliteal lymph nodes were analyzed, and the expression of **a** CD25 and **b** CD69 was quantified (*n* = 6 mice). Data are means±SEM analyzed by unpaired Student’s *t*-test **c** Splenocytes from CD45.1^+^ WT/OTII and CD45.2^+^ *Lmna*^*−/−*^/OTII mice were mixed, stained with cell violet, and cultured in the presence of OVA for 2 days. CD4^+^ T-cell proliferation was quantified and the number of cell divisions is shown in the graph. Data are means±SEM of six independent experiments analyzed by two-way ANOVA with Bonferroni’s multiple comparison test. **d**, **e** CD4^+^ T-cells from CD45.1^+^ WT and CD45.2^+^ *Lmna*^*−/−*^ mice were mixed 1:1 and adoptively transferred to CD45.1/CD45.2 WT mice. Recipient mice were then intraperitoneally infected with VACV for 7 days. The peritoneal exudates and mesenteric lymph nodes were analyzed, and the percentage of **d** Ki67^+^ or **e** PCNA^+^ CD4^+^ T-cells was quantified (*n* = 5 mice from 2 independent experiments) Data are means±SEM analyzed by unpaired Student’s *t*-test. **f** CD45.1^+^ WT recipient mice were transplanted with a 1:1 mix CD45.1^+^ WT and CD45.2^+^ *Lmna*^*−/−*^ bone marrow and infected intraperitoneally with VACV. Mice were injected with BrdU, and 24 h later the percentage of BrdU^+^ CD4^+^ T-cells was quantified in mesenteric lymph nodes, peritoneal exudate, and spleen (*n* = 3 mice from 1 representative experiment) Data are means±SEM analyzed by paired Student’s *t*-test. **g** CD4^+^ T-cells from CD45.2^+^ WT or CD45.2^+^ *Lmna*^*−/−*^ mice were mixed 1:1 and adoptively transferred to CD45.1^+^/CD45.2^+^ WT mice. Recipient mice were infected intraperitoneally with VACV for 6 days and injected with BrdU. After 24 h, the percentage of BrdU^+^ CD4^+^ T-cells was quantified in the peritoneal exudate and mesenteric lymph nodes (*n* = 5 and 5 mice). Data are means±SEM analyzed by paired Student’s *t*-test. ***P < 0.001

Reduced Th1 cell polarization from *Lmna*^*−/−*^ CD4^+^ T-cells could be due to impaired T-cell activation, which is enhanced by lamin A in vitro^3^. We therefore analyzed CD4^+^ T-cell activation in response to VACV infection in the absence of lamin A/C expression. A 1:1 mix of naive CD4^+^ T-cells from *Lmna*^*−/−*^/OTII and WT/OTII mice was adoptively transferred into WT recipient mice before intradermal infection with VACV-OVA. Surface expression of the T-cell activation markers CD25 and CD69 was measured 48 h later (Fig. 4a, b). Accordingly, compared with WT cells, *Lmna*^*−/−*^ CD4^+^ T-cells in draining lymph nodes expressed less CD25 and CD69.

Next, we investigated whether lamin A/C could regulate T-cell proliferation, which occurs after antigen recognition and activation. However, proliferation after in vitro activation with anti-CD3 and anti-CD28 was similar in *Lmna*^*−/−*^ and WT CD4^+^ T-cells (Fig. 4c). Similarly, after VACV infection, WT and *Lmna*^*−/−*^ mice had similar levels of proliferating CD4^+^ T-cells, as shown by Ki67 expression (Fig. 4d), proliferating cell nuclear antigen (PCNA) expression (Fig. 4e), and 5-bromo-2-deoxyuridine (BrdU) incorporation (Fig. 4f–g). These experiments show that A-type lamins regulate T-cell activation without affecting cell proliferation.

### Lamin A/C enhances T-bet levels

**Fig. 5:**
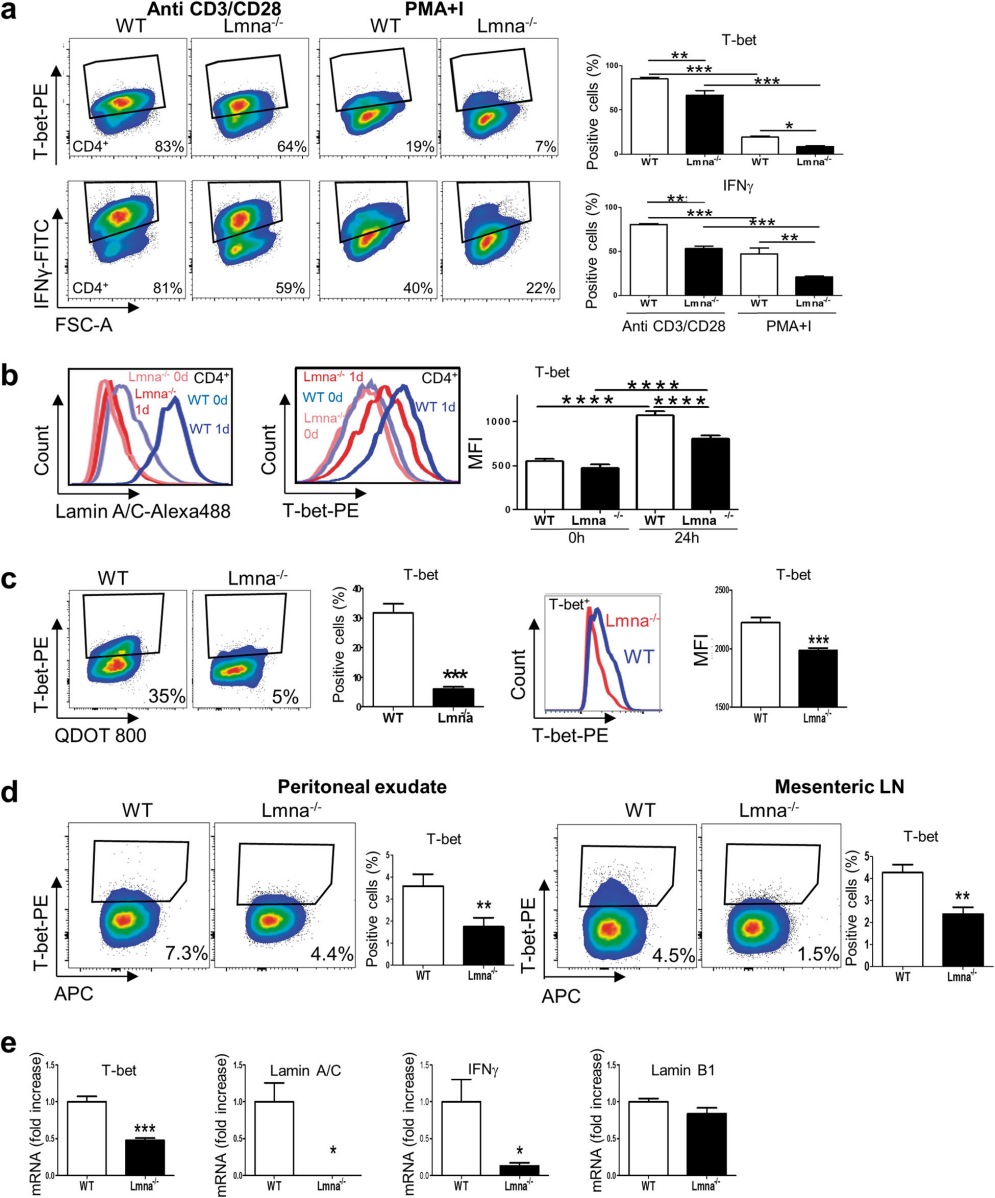
*Lmna*^−/−^ CD4^+^ T-cells show impaired in vitro and in vivo T-bet expression **a** Percentage of IFNγ^+^ and T-bet^+^ cells after stimulation of CD4^+^ T-cells from WT and *Lmna*^*−/−*^ mice with anti-CD3/CD28 antibodies or PMA plus ionomycin for 4 days in the presence of IL-2 (Data are means±SEM of at least 2 independent experiments analyzed by one-way ANOVA with Bonferroni’s multiple comparison test. **b** T-bet and lamin A/C expression in WT and *Lmna*^*−/−*^ CD4^+^ T-cells 48 h after stimulation with anti-CD3/CD28 antibodies (*n* = 12 and 12 mice). Data are means±SEM analyzed by one-way ANOVA with Bonferroni’s multiple comparison test **c** Percentage of T-bet^+^ cells after stimulation of WT and *Lmna*^*−/−*^ CD4^+^ T-cells with anti-CD3/CD28 for 7 days in the presence of Th1 polarizing antibodies and cytokines (*n* = 5 and 6 mice from 2 independent experiments). Data are means±SEM analyzed by unpaired Student’s *t*-test **d** Percentage of T-bet^+^ CD4^+^ T-cells in peritoneal exudates and mesenteric lymph nodes of WT recipient mice reconstituted with a 1:1 mix of WT and *Lmna*^*−/−*^ bone marrow and intraperitoneally infected with VACV for 6 days (*n* = 4 and 4 mice from 2 independent experiments). Data are means±SEM analyzed by paired Student’s *t*-test **e** RT-qPCR analysis of the indicated genes in WT and *Lmna*^*−/−*^ CD4^+^ T-cells stimulated in vitro with anti-CD3/CD28 antibodies (*n* = pools of 3 mice). Data are means±SEM from 3 independent experiments analyzed by unpaired Student’s *t*-test *P < 0.05; **P < 0.01; ***P < 0.001; ****P < 0.0001

The master regulator of Th1 differentiation is T-bet. Analysis of T-bet expression in CD4^+^ T-cells revealed that deficiency in A-type lamins reduced both the percentage of T-bet^+^ cells and T-bet expression levels in anti-CD3/CD28-stimulated CD4^+^ T-cells (Fig. 5a, b). In agreement with the effect on Th1 differentiation, lamin A/C deficiency also diminished the percentage of IFNγ^+^CD4^+^ T-cells (Fig. 5a). Similar results were obtained after stimulation with anti-CD3/CD28 antibodies in the presence of Th1-differentiation cytokines (Fig. 5c). Moreover, lamin A/C expression was also important for T-bet expression in vivo after Th1 differentiation triggered by VACV infection (Fig. 5d). Low T-bet protein expression in *Lmna*^*−/−*^ T-cells was accompanied by lower mRNA expression in vitro (Fig. 5e), suggesting regulation at the level of mRNA synthesis.

A-type lamins regulate TCR clustering and subsequent downstream signaling upon T-cell activation^3^. However, altered Th1 differentiation in the absence of lamin A/C was not exclusively related to differences in TCR clustering, since the percentage of IFNγ^+^ and T-bet^+^ *Lmna*^*−/−*^ CD4^+^ T-cells was also lower after TCR-independent T-cell stimulation with phorbol myristate acetate (PMA) and ionomycin (Fig. 5a).

### Lamin A/C regulates Th1 immunity against pathogen infection

**Fig. 6:**
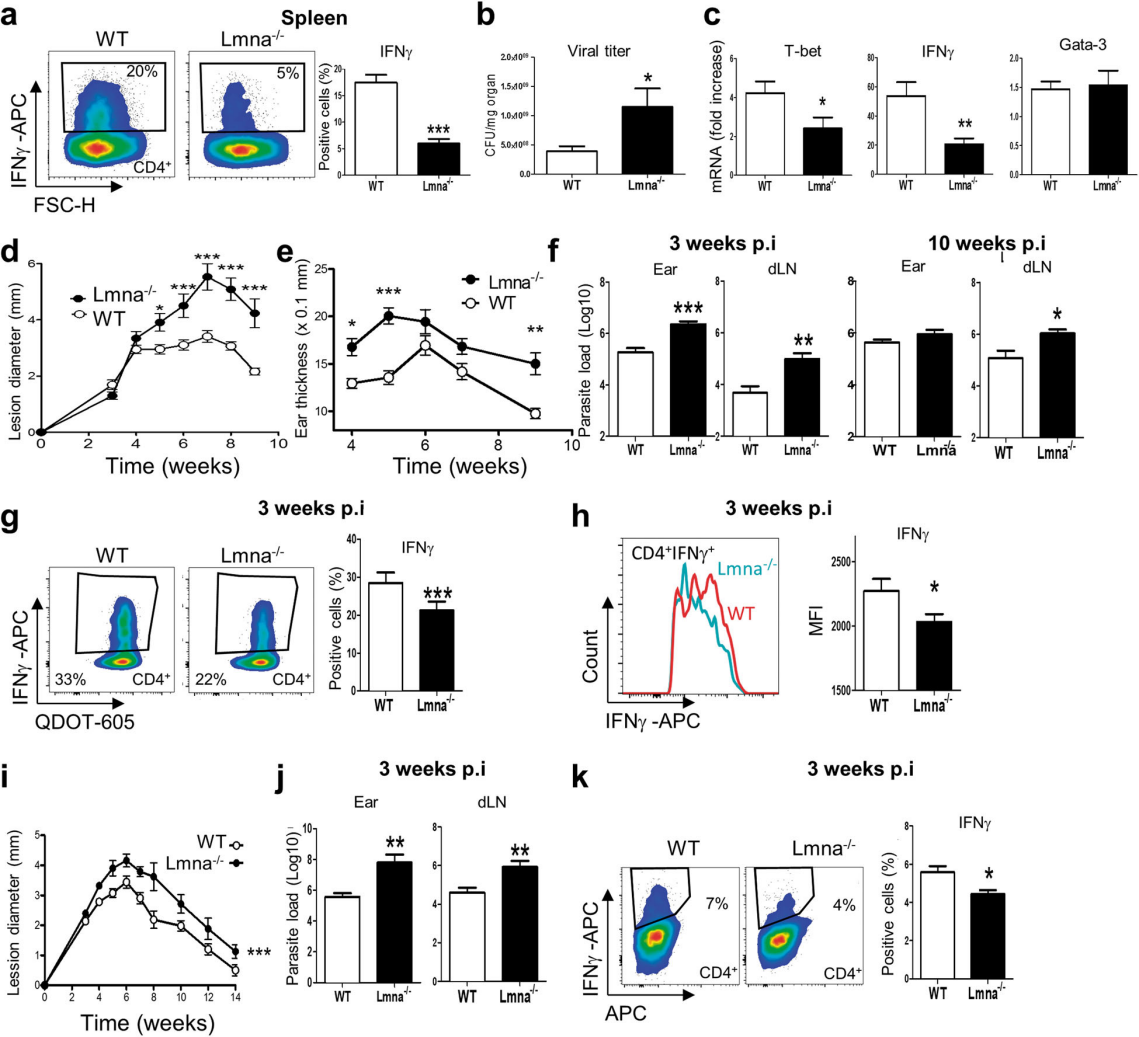
Lamin A/C deficiency enhances susceptibility to VACV and *Leishmania major* **a–c** *Rag1*^*−/−*^ mice were inoculated intravenously with naive WT or *Lmna*^*−/−*^ CD4^+^ T-cells and infected with VACV by tail scarification. After 10 days, animals were analyzed to determine **a** the percentage of IFNγ^+^ CD4^+^ T-cells in the spleen (*n* = 7 and 5 mice), **b** the viral titers in the tail (*n* = 5 and 5 mice), and **c** the mRNA levels of T-bet (Tbx21), IFNγ, and Gata3 in the gut (*n* = 7 and 8 mice). **d**–**h** WT mice were adoptively transferred with WT or *Lmna*^*−/−*^ bone marrow. Two months after reconstitution, *Leishmania major* promastigotes were injected intradermally in the ears (*n* = 8–9 mice). Lesion size **d** and ear thickness **e** were quantified at the indicated times. **f** Parasite burden in ears and draining lymph nodes was measured 3 and 10 weeks after infection. **g**,** h** The percentages of **g** IFNγ^+^ CD4^+^ T-cells and **h** the CD4^+^ T-cell IFNγ content were quantified in the ears 3 weeks after infection. **i**–**k** WT and CD4-CRE *Lmna*^flox/flox^ mice were injected intradermally in the ears with *Leishmania major* promastigotes (*n* = 9 and 9 mice). **i** Lesion size at the indicated times. **j** Parasite burden in the ears and draining lymph nodes at 3 weeks postinfection. **k** Percentage of IFNγ^+^ CD4^+^ T-cells in the ears. Data analysis in **a**–**c**, **f**–**h**, **k**: unpaired Student’s *t*-test; **d**–**e**, **i**: two-way ANOVA with Bonferroni’s post-hoc multiple comparison test. *P < 0.05; **P < 0.01; ***P < 0.001

T-bet and IFNγ play major roles during in vivo Th1 responses against pathogens^11,15,16^. The low expression of these factors in *Lmna*^*−/−*^ CD4^+^ T-cells prompted us to investigate the role of lamin A/C in Th1-mediated pathogen clearance in vivo. *Rag1*^*−/−*^mice lacking T- and B-cells were adoptively transferred with naive CD25^-^CD4^+^ T-cells from WT or *Lmna*^*−/−*^ mice. Mice were infected with VACV by tail scarification. Without CD4^+^ T-cell adoptive transfer, 100% of *Rag1*^*−/−*^ mice die 12 days after VACV infection; CD4^+^ T-cell adoptive transfer extends lifespan by accelerating viral clearance at least in part through a mechanism mediated by Th1 cells^17^. In our experiments, *Rag1*^*−/−*^ mice adoptively transferred with CD25^-^CD4^+^ *Lmna*^*−/−*^ T-cells were more susceptible to primary VACV infection, as indicated by an elevated viral titer and a reduced Th1 response in the spleen (Fig. 6a, b). Moreover, IFNγ and T-bet mRNA expression was reduced in adoptively transferred mice, whereas the Th2 transcription factor GATA3 was unaffected (Fig. 6c). Viral clearance requires the efficient coordination of multiple immune effector mechanisms^11^. The role of lamin A/C in viral clearance seems to be CD4^+^ T-cell dependent, since only CD4^+^ T-cells were adoptively transferred to *Rag1*^*−/−*^ mice before VACV infection. Effector CD4^+^ T-cells have been shown to respond to viral pathogens through two mechanisms: cytokine production (mostly IFNγ and tumor necrosis factor-α (TNFα)) and a direct cytolytic activity mediated by perforin and FAS (also known as CD95)^11^. CD4^+^ T-cell cytotoxic activity does not require Th1 cell polarization but is dependent on the expression of the transcription factors T-bet, eomesodermin (Eomes), and Blimp-1, which are crucial for their development in vivo through the induction of granzyme B (GzmB) and perforin 1 (Prf1)^18,19^. Interestingly, *Rag1*^*−/−*^ mice adoptively transferred with *Lmna*^*−/−*^ CD4^+^ T-cells and scarified with VACV had lower levels of IFNγ mRNA in the large intestine than mice receiving WT CD4^+^ T-cells (Fig. 6c). In addition, we found that lamin A/C expression in CD4^+^ T-cells differentiated to Th1 in vitro positively regulates the mRNA expression of eomes, Blimp1, GzmB, and Prf1 but not granzyme A (GzmA) (Figure S3). Lamin A/C also regulates CD4^+^ T-cell cytoxicity in vivo, since VACV-OVA-generated *Lmna*^*−/−*^ Th1 cells were less efficient to kill OVA-loaded B220^+^MHCII^+^ B cells, after being adoptively transferred to *Rag1*^*−/−*^ mice (Figure S4).

We also analyzed the importance of lamin A/C expression for Th1 responses against the intracellular parasite *L. major*^16^. Mice on the C57BL/6 background control *L. major* infection by developing a protective Th1 response^20^. C57BL/6 mice lacking lamin A/C in the whole immune system (Fig. 6d–h) or specifically in T-cells (Fig. 6i–k) developed progressively larger and non-healing lesions (Fig. 6d, e, i) after inoculation of the ear dermis with a low dose of *L. major* parasites. This effect correlated with a higher parasite burden in the infection site and in draining lymph nodes after 3 weeks (Fig. 6f, j). At this time, *Lmna*^*−/−*^ mice showed a reduced percentage of IFNγ^+^ CD4^+^ T-cells (Fig. 6g, k), with less IFNγ production (Fig. 6h). These results further support the role of lamin A/C in Th1 responses against pathogens.

## Discussion

The role of nuclear envelope proteins in the regulation of immune homeostasis remains largely unknown^21^. Similarly, little is known about the molecular signals that switch immune function from protective to tissue damaging. Adaptive immunity is finely regulated by the interplay between immune regulatory mechanisms and effector T-cell responses. T-cells play central roles in orchestrating the protection against diverse microbial pathogens^4^. Many studies have investigated membrane mediators important for T-cell fate decision, while others have assessed the role of cytoplasmic proteins; however, the importance of nuclear envelope proteins for naive T-cell differentiation remained overlooked. Despite studies characterizing signaling pathways from the membrane to the nucleus, there is a lack of information about signals arising in the nuclear interior and the role of nuclear proteins in mediating the transmission of information to the cytoplasm. It has been previously shown that intrinsic lamin A/C deficiency does not affect T- and B-cell development, driving the generation of functional and self-MHC-restricted CD4^+^ and CD8^+^ T-cells^10^. We previously demonstrated that transient lamin A/C expression in CD4^+^ T-cells after antigen recognition contributes to T-cell activation^3^. In the present study, we have identified the nuclear envelope protein lamin A/C as a crucial driver of Th1 polarization, both in vitro and in vivo.

Lamin A/C is expressed upon T-cell activation, and its deficiency downregulates the transcription factor T-bet, thereby impairing naive CD4^+^ T-cell differentiation toward the Th1 phenotype. Strikingly, this effect is not counterbalanced by an increase in Th2 differentiation. Distinct signaling pathways can drive T-cell differentiation^22^. In response to TCR activation and mTOR costimulation, Akt modulates transcription of T-bet but not Gata3, thus affecting Th1 but not Th2 differentiation. In contrast, PKC-θ activation yields the opposite result^22^. Lamin A/C is an important regulator of gene transcription during adipose tissue differentiation^23^ and controls the phosphorylation of signaling molecules in other cellular contexts^1,24^. Indeed, we previously showed that lamin A/C binds to ERK1/2 and c-Fos, regulating AP-1 transcription factor target genes^24,25^ and that lamin A/C controls CD4^+^ T-cell activation through ERK1/2 signaling^3^. Interestingly, the formation of c-Fos/c-Jun dimers can potentiate Th1 differentiation^26^. Lamin A/C thus seems to specifically regulate pathways involved in Th1 differentiation, directly modulating the transcription of Th1-related genes. This process occurs in a TCR-dependent manner, although lamin A/C can also regulate signaling downstream the TCR, as observed after stimulation with PMA plus ionomycin.

The Th fate of antigen-engaged T-cells depends on many factors, including the type of antigen encountered, the TCR signal intensity, the duration of antigen presentation, and the local cytokine milieu^4,6–8,26,27^. Th1 differentiation is favored by stronger T-cell activation signals and longer T–DC cell–cell interactions^8,26^. Lamin A/C enhances T-cell activation by increasing TCR signal strength and promoting T–DC interaction^3^. Weaker TCR signal intensity in the absence of lamin A/C could account for the observed reduction in CD4^+^ T-cell activation triggered by VACV infection. However, this cannot fully explain the differences in Th1 vs. Th2 differentiation in lamin A/C-deficient T-cells, indicating that additional factors are involved. Regarding the cytokine milieu, IL-2, IL-12, and IFNγ are known to facilitate Th1 differentiation, whereas IL-4 favors Th2 differentiation. Moreover, some cytokines promote differentiation to a specific Th phenotype without affecting others. For example, IL-33 enhances Th1 but not Th2 polarization in the presence of IL-12^28^. Lamin A/C expression could regulate the production and release of cytokines important for Th1 phenotype. In this regard, we find that lamin A/C deficiency reduces membrane expression of the IL-2 receptor CD25 in CD4^+^ T-cells activated by VACV infection in vivo. Incubation of CD4^+^ T-cells with IL-2 or Th1 polarizing cytokines during in vitro differentiation did not rescue the impaired *Lmna*^*−/−*^ Th1 differentiation, indicating that intracellular signals driven by the nuclear envelope protein lamin A/C directly regulate Th1 differentiation.

Viral clearance requires the efficient coordination of multiple immune effector mechanisms^11^. We show that lamin A/C deficiency in CD4^+^ T-cells results in a defective Th1 differentiation, impairing the response against VACV and delaying viral clearance. These results suggest that lamin A/C is an essential component of the immune response against cytopathic viruses. Remarkably, the role of lamin A/C in viral clearance seems to be CD4^+^ T-cell dependent, since only CD4^+^ T-cells were adoptively transferred to T- and B-cell-deficient *Rag1*^*−/−*^ mice before mouse infection with VACV. Accordingly, in humans CD4^+^ T-cells are critical for antiviral immunity and control of VACV replication^12^. CD4^+^ T-cells can also regulate innate immune responses that mediate viral clearance^11^, which is independent on B-cells and CD8^+^ T-cells, and might be important for appropriate CD4^+^ T-cell responses against VACV^17^. Interestingly, *Lmna*^*−/−*^ CD4^+^ T-cells show reduced cytotoxic activity in vivo and impaired in vitro mRNA expression of transcription factors involved in the regulation of CD4^+^ T-cell cytotoxicity, like eomes, Blimp1, GzmB, and Prf1.

The protozoan parasite *L. major* cause cutaneous leishmaniasis^29^, usually provoking local self-healing skin lesions in humans. However, the parasite can cause prolonged non-healing skin ulcers with extensive tissue destruction and become systemically distributed^29^. C57BL/6 mice develop protective immune responses against *L. major*. We show that WT recipient C57BL/6 mice reconstituted with *Lmna*^*−/−*^ bone marrow develop poorer Th1 responses against this parasite, with worse pathogen clearance than recipients of WT cells. The immune response against *L. major* involves multiple cell types. Importantly, our results clearly demonstrate that lack of lamin A/C specifically in T-cells impaired healing of ear lesions. C57BL/6 mice develop Th1-dependent responses to *L. major*, and following a high *L. major* dose the lesions heal in the absence of CD8^+^ T-cells^30,31^. Moreover, cytolytic CD8^+^ T-cells are pathogenic when recruited to *Leishmania* lesions in large numbers^32^. Therefore, impaired responses observed in Lmna^flox/flox^×CD4-CRE mice likely associate with lamin A/C-deficient CD4^+^ T-cells. The early immune response determines whether *L. major* skin lesions will be self-healing or chronic. C57BL/6 mice develop CD4^+^ Th1 cell-mediated resistance, whereas other mouse strains develop CD4^+^ Th2 responses and are extremely susceptible to infection^33^. Therefore, CD4^+^ Th1 cells are crucial for reducing *Leishmani*a infection^34^, producing IFNγ, IL-2, and TNFα locally and leading to macrophage activation and parasite elimination. Our experiments show a diminished percentage of IFNγ-producing CD4^+^ T-cells and reduced IFNγ production in *Lmna*^*−/−*^ mice. IFNγ secretion by CD4^+^ T-cells induces macrophages to produce nitric oxide and TNF, thus controlling *L. major* infection^33,35^. This mechanism could explain the diminished pathogen clearance observed in the absence of lamin A/C. Importantly, we show that lamin A/C in CD4^+^ T-cell is an important mediator of the immune control against this pathogen.

Our study reveals a novel role for lamin A/C as a regulator of T-cell differentiation, controlling the maintenance of Th1 populations in response to pathogen infections. These data contribute to the understanding of the molecular mechanisms driving CD4^+^ T-cell responses and suggest that strategies to modulate T-cell function could provide a route toward therapeutic immunization and long-lasting protection.

## Materials and methods

### Mice

*Lmna*^*−/−*^ mice have been described previously^36^. C57BL/6-Tg (TcraTcrb)425Cbn/J mice (OTII mice) express a TCR specific for the OVA peptide (amino acid residues 323–339) in the context of I-Ab and the CD4-CRE and were obtained from the Jackson Laboratory (stock number 004194 and 017336, respectively). C57BL/6-CD45.2^+^*Lmna*^*−/−*^ OTII mice were generated by crossing C57BL/6-CD45.2^+^ OTII mice with C57BL/6-CD45.2^+^*Lmna*^*−/−*^ mice. C57BL/6-CD45.1^+^CD45.2^+^ WT mice were generated by crossing C57BL/6-CD45.2^+^ mice with C57BL/6-CD45.1^+^ mice. C57BL/6-CD45.1^+^ and C57BL/6-CD45.1^+^CD45.2^+^ WT mice were used as recipients for adoptive transfer. C57BL/6-CD45.1^+^CD45.2^+^/OTII WT mice were generated by crossing C57BL/6-CD45.2^+^/OTII mice with C57BL/6-CD45.1^+^/OTII mice. *Lmna*^flox/flox^ mice were kindly provided by Y. Zheng^37^. C57BL/6*Lmna*^flox/flox^CD4-CRE mice were generated by crossing C57BL/6-CD4-CRE with C57BL/6-*Lmna*^flox/flox^ mice. C57BL/6 transgenic β-actin DsRed mice (Tg(ACTB-DsRed*MST)1Nagy/J; The Jackson Laboratory) was also used. All mice were bred at the CNIC in specific pathogen-free conditions.

Animal experiments were approved by the local ethics committee and the Spanish Ministry of Agriculture and Fisheries, Food and Environment. All animal procedures conformed to EU Directive 86/609/EEC and Recommendation 2007/526/EC regarding the protection of animals used for experimental and other scientific purposes, enforced in Spanish law under Real Decreto 1201/2005.

### Antibodies and reagents

Anti-lamin-A/C (n-18), anti-p-ERK1/2, anti-PCNA, and PE-conjugated anti-lamin A/C were obtained from Santa Cruz Biotechnology. Alexa Fluor 488-conjugated anti-lamin-A/C was obtained from Cell Signaling. Anti-CD3, anti-CD28, fluorescein isothiocyanate (FITC)-conjugated-CD45.1, V450-conjugated anti-CD45.1, PerCPCy5.5-conjugated anti-CD45.1, PerCPCy5.5-conjugated anti-CD45.2, APC-conjugated anti-CD45.1, v450-conjugated anti-CD45.2, FITC-conjugated anti-CD45.2, APC-conjugated anti-IFNγ, and V450-conjugated anti-CD4 were from Tonbo Bioscience. PECy5-conjugated anti-CD45.1, PECy7-conjugated anti-CD45.1, PE-conjugated anti-Tbet, FITC-conjugate anti-CD8, APC-conjugated anti-CD69, APC-conjugated anti-CD25, PE-conjugated anti-T-bet, FITC-conjugated anti-Ki67, PE-conjugated anti-IL-4, and biotinylated antibodies against B220, CD19, MHCII, CD11c, CD11b, CD44, CD49b, IgM CD25, and CD8α were from BD Biosciences.

### T-cell stimulation and polarization

CD4^+^CD25^−^ T-cells from spleens were purified by negative selection on separation columns (Miltenyi Biotec) after labeling the cells with a cocktail of biotinylated antibodies against B220, CD19, MHCII, CD11c, CD11b, CD44, CD25, CD49b, IgM, and CD8α and a solution containing streptavidin-bound magnetic microbeads (Miltenyi Biotec). For polarizing experiments, CD4^+^CD25^−^ T-cells from WT/OTII and *Lmna*^*−/−*^/OTII mice were stimulated with irradiated autologous WT APCs previously incubated with OVA peptide (10 μg/ml) for 30 min at 37 °C. CD4^+^CD25^−^ T-cells from WT and *Lmna*^*−/−*^ mice were stimulated with plate-bound anti-CD3 (10 μg/ml) and soluble anti-CD28 antibodies (2 μg/ml).

Polarizing conditions were as follows: for Th1 polarization: IL-12 (10 ng/ml) and anti-IL-4 (4 μg/ml), with only IL-2 (10 ng/ml) during the rest period; for Th2 polarization: IL-4 (10 ng/ml) and anti-IFNγ (4 μg/ml), with only IL-2 (10 ng/ml) during the rest period. Antibodies and cytokines were from BD bioscience and Tonbo, respectively. Cells were cultured in RPMI-1640 medium (GIBCO) for the indicated times.

### Adoptive transfer

Splenic CD4^+^CD25^-^ T-cells from CD45.2^+^ *Lmna*^*−/−*^, CD45.1^+^CD45.2^+^ WT, or CD45.2^+^ WT mice were isolated by negative selection on MACS separation columns (Miltenyi Biotec) after labeling the cells with a cocktail of biotinylated antibodies against B220, CD19, MHCII, CD11c, CD11b, CD44, CD25, CD49b, IgM, and CD8α, as well as with a solution containing streptavidin-bound magnetic microbeads (Miltenyi Biotec). Adoptive transfer experiments were performed by inoculating CD45.1^+^ or CD45.1^+^CD45.2^+^ WT recipient mice with 1×10^6^ isolated CD4^+^ T-cells. In some experiments, CD45.1^+^CD45.2^+^ WT recipient mice were inoculated with a 1:1 mix of 2 × 10^6^ CD25−CD4^+^ T-cells obtained from the spleens of CD45.2^+^ *Lmna*^*−/−*^ and CD45.1^+^ WT mice. In VACV experiments, adoptive cell transfer was performed 24 h before inoculation with the VACV virus^38^. Similar adoptive transfers were performed with CD4^+^ T-cells isolated from CD45.1^+^CD45.2^+^ WT/OTII, CD45.1^+^ WT/OTII, or CD45.2^+^ Lmna^*−/−*^ mice.

### Generation of BMDCs

BMDCs were generated as described in ref. 39. BMDCs were obtained from bone marrow cell suspensions after culture on non-treated cell culture dishes in complete RPMI 1640 medium supplemented with 10% fetal bovine serum (FBS), 2 mM L-glutamine, 100 mg/ml penicillin, 100 mg/ml streptomycin, 50 mM 2-ME, and 20 ng/ml GM-CSF (PeproTech, London, UK). Cells were collected at day 9 and BMDCs were isolated as CD11c+ MHC-II+ Ly6G− cells. Maturation was induced after overnight incubation with LPS from *Escherichia coli* O111:B4 (1 μg/ml; Sigma-Aldrich).

### Vaccinia virus, scarification assays, and viral titration in organs

The VACV strain Western Reserve (WR, ATCC number VR-1354) was a gift from Jonathan W. Yewdell and Jack R. Bennink (NIH, Bethesda, Maryland, USA). VACV was propagated in BSC-40 cells and purified by sucrose gradient ultracentrifugation. WT or OVA-expressing VACV (1 × 10^6^ plaque-forming units) were administered by intraperitoneal injection or intradermal injection in the footpad^38^.

Animals were anesthetized with an intraperitoneal injection of Ketamine (100 mg/kg body weight) and Xylazine (10 mg/kg) diluted in sterile phosphate-buffered saline (PBS). Thirty 1 cm scarifications were made with a 26 G syringe along the base of the tail, avoiding bleeding^40^. Then 10 ml of PBS containing 10^7^ VACV plaque-forming units (p.f.u.) were added to the area and allowed to air dry. For mock infection only, PBS was added.

Animals were euthanized and the tail aseptically removed, weighed, frozen at −80 °C, and stored at −80 °C until use. The samples were homogenized with a Tissue Ruptor (QIAGEN, USA) in 0.5 ml Dulbecco’s modified Eagle’s medium (DMEM) containing 100 U/ml penicillin and 100 µg/ml streptomycin. The homogenates were sonicated for 3 min at 40% amplitude, freeze-thawed twice (−80 °C/37 °C), sonicated again under the same conditions, and then serially diluted in DMEM without FBS. To quantify p.f.u., the dilutions were added to monolayers of CV-1 cells seeded on 24-well plates. The cells were preincubated for 1 day at 37 °C and 5% CO_2_, the dilutions (0.2 ml) were added to each well, and the cells were further incubated under the same conditions for 1 h. After this, 0.5 ml DMEM containing 0.5% FBS were added to each well. After 24 h, the cells were stained for 5 min with crystal violet solution (0.5% crystal violet, 10% ethanol, and 1% paraformaldehyde) and washed again. Viral plaques were counted and plaque number was multiplied by the reciprocal of sample dilution and converted to p.f.u./g.^17^

### *Leishmania* parasites, preparation, inoculation, and quantification

In vivo experiments were carried out using *L. major* Friedlin strain FV1 (MHOM/IL/80/Friedlin), generously provided by Dr. D. Sacks (NIH)^20^. For *Leishmania* challenge, parasites were kept in a virulent state by passage in mice. Parasites were cultured and differentiated as described^41^. Mice were infected by i.d. injection of 10^3^ or 5 × 10^4^ metacyclic *L. major* promastigotes into the dermis of both ears. Lesion size in the ear was determined with digital callipers (Duratool)^20^. The limiting dilution assay was used to determine parasite number^41^. Parasite load was expressed as the number of parasites in the whole organ.

### Bone marrow transplantation

WT recipient mice received 13 Gy of total body irradiation administered in two treatments from a ^137^Cs source. Bone marrow cells from CD45.2^+^ WT or CD45.2^+^ *Lmna*^*−/−*^ mice were transplanted into CD45.1^+^ WT recipients by injection into the retro-orbital sinus immediately after irradiation. A mix 1:1 of bone marrow cells from CD45.1^+^ WT and CD45.2^+^ *Lmna*^*−/−*^ mice were transplanted into CD45.1^+^CD45.2^+^ WT recipients by injection into the retro-orbital sinus immediately after irradiation. Approximately 8 weeks after transplantation, the chimeric condition of the mice was assessed by flow cytometric analysis of blood cells stained with a combination of fluorescently labeled anti-CD45.1 and anti-CD45.2 antibodies to detect T-cells from donor and from recipient mice, which confirmed that >90% of the cells analyzed were derived from the transplanted bone marrow cells

### Flow cytometry

CD4^+^ T-cells were stimulated with PMA (20 ng/ml) (Sigma Aldrich) plus ionomycin (1 μg/ml) (Sigma Aldrich) for 6 h. Brefeldin A (Sigma Aldrich) was added for the last 2 h to allow intracellular cytokine accumulation. Surface antigens were stained with antibodies, then fixed and made permeable with either a Cytofix/Cytoperm kit (BD Pharmingen), then intracellular cytokines and transcription factors in cells were stained. Data were acquired on FACSCantoII or LSRFortessa flow cytometers (BD Biosciences) and analyzed with the BD FACSDIVA (BD Biosciences) or FlowJo (Treestar Inc) software.

### Reverse transcription–quantitative PCR

Total RNA was isolated with Qiazol Lysis Reagent (Qiagen) and isopropanol precipitation or with the RNeasy Mini kit (Qiagen), according to the manufacturer’s instructions. RNA concentration and purity were assessed from the ratio of absorbances at 260 and 280 nm. Complementary DNA (cDNA) was synthesized from total RNA (0.1–1 µg per reaction) with the High Capacity cDNA Reverse Transcription Kit (Applied Biosystems) using random primers and RNase Inhibitor, according to the manufacturer’s protocol. Quantitative PCR was performed with the ABI PRISM 7900HT Sequence Detection System (Applied Biosystems) using the PCR Power SYBR Green PCR Master Mix (Applied Biosystems), with technical triplicates.

The sequences of RT-qPCR primers were as follows: T-Bet-Forward 5′-GAAAGGCAGAAGGCAGCAT-3′; T-Bet-Reverse 5′-GAGCTTTAGCTTCCCAAATGAA-3′; Hprt1-Forward 5′-CCTAAGATGAGCGCAAGTTGAA-3′ and Hprt1-Reverse 5′-CCACAGGACTAGAACACCTGCTAA-3′; *GzmA*-Forward 5′-GACTGCTGCCCACTGTAACG-3′; *GzmA*-Reverse 5′-TCAATATCTGTTGTTCTGGCTCCTTA-3′; *GzmB-*Forward 5′-TGTCTCTGGCCTCCAGGACAA-3′; *GzmB*-Reverse 5′- CTCAGGCTGCTGATCCTTGATCGA-3′; *Prf1*-Forward 5′- GCGTCTCCAGTGAATACAAAG-3′; *Prf1-*Reverse 5′- TACTTCGACGTGACGCT-3′; *Eomes*-Forward 5′-GCCTACCAAAACACGGATA-3′; *Eomes*-Reverse 5′-TCTGTTGGGGTGAGAGGAG-3′; *Ifng*-Forward 5′- TGGCTCTGCAGGATTTTCATG-3′; *Ifng*-Reverse 5′- TCAAGTGGCATAGATGTGGAAGAA-3′; *Blimp-1*-Forward 5′- ACACACAGGAGAGAAGCCACATGA-3′; *Blimp-1*-Reverse 5′- TCGAAGGTGGGTCTTGAGATTGCT-3′; *gapdh*-Forward 5′-CTACACTGAGGACCAGGTTGTC-3′; *gapdh*-Reverse 5′-GGTCTGGGATGGAAATTGTG-3′. *Lmnb1*-Forward 5′-CAACTGACCTCATCTGGAAGAAC 3′; *Lmnb1*-Reverse 5′-TAAGACTGTGCTTCTCTGAGC-3′; *Lmna*-Forward-5′-TGAGTACAACCTGCGCTCAC-3′; and *Lmna*-Reverse: 5′-TGACTAGGTTGTCCCCGAAG-3′. The extent of expression of a gene of interest was analyzed by the comparative Ct method with the Biogazelle qBasePLUS software using as internal control the housekeeping genes GAPDH (glyceraldehyde-3-phosphate dehydrogenase) and HPRT1 (hypoxanthine phosphoribosyltransferase 1). Results are represented as fold change relative to control conditions.

### Proliferation assays

Twenty-four hours before the sacrifice of the mice, a single dose of 1 mg BrdU (BD Pharmingen) was injected intraperitoneally (i.p.). To assess BrdU incorporation, mesenteric lymph nodes, peritoneal exudate, and spleen were stained for CD4, fixed, and permeabilized using the BD BrdU Flow Kit (BD Pharmigen) according to the manufacturer’s instructions. Cells were incubated at 37 °C for 60 min in 30 µg of DNase, followed by staining with anti-BrdU-FITC for 40 min, washed, and analyzed by flow cytometry. Peritoneal exudate and mesenteric lymph node cells were fixed and permeabilized using Foxp3 fixation/permeabilization buffer (BD Pharmingen) and stained for PCNA-FITC and Ki67-FITC.

### In vivo cytotoxicity assays

Splenic CD45.2^+^WT or CD45.2^+^*Lmna*^*−/−*^ CD4^+^/OTII T-cells were isolated as described in the adoptive transfer section. Recipient *Rag1*^*−/−*^ mice were inoculated intravenously with 2 × 10^6^ of either naive CD45.2/WT or CD45.2/*Lmna*^*−/−*^ CD4^+^/OTII T-cells and infected with VACV-OVA i.p. After 5 days, splenocytes from CD45.2/WT/dsRED and CD45.1/WT mice were isolated and loaded or not with OVA OTII peptide, respectively. An approximately 1:1 mix of 5 × 10^6^ CD45.2/WT/dsRED and CD45.1/WT splenocytes was inoculated intravenously in the recipient *Rag1*^*−/−*^ mice, which were previously inoculated and vaccinia infected. After 16 h, animals were analyzed to determine the killing capacity of CD4/OTII cells in the spleen by flow cytometry. Killing capacity was determined as the ratio between CD45.2/WT/dsRED/+OVA and CD45.1/WT/-OVA cells and the percentage of CD4/OTII T-cells. Mice adoptively transferred with CD4/OTII cells only and mice transferred with splenocytes in the absence of CD4/OTII cells were used as controls.

### Statistical analysis

Statistical analyses were performed with Prism GraphPad or Microsoft Office Excel. Unless otherwise stated, statistical significance was calculated by two-tailed Student’s *t*-test. When specified, one-way analysis of variance (ANOVA) or two-way ANOVA with Bonferroni’s post-hoc multiple comparison test was used. Significance of differences was calculated as follows: *P < 0.05, **P < 0.01, ***P < 0.001, and **** *P* > 0.0001.

## Electronic supplementary material

Figure S1

Figure S2

Figure S3

Figure S4

Supplementary Information

## References

[1] Andres V, Gonzalez JM (2009). Role of A-type lamins in signaling, transcription, and chromatin organization. J. Cell Biol..

[2] Broers JL, Ramaekers FC, Bonne G, Yaou RB, Hutchison CJ (2006). Nuclear lamins: laminopathies and their role in premature ageing. Physiol. Rev..

[3] Gonzalez-Granado JM (2014). Nuclear envelope lamin-A couples actin dynamics with immunological synapse architecture and T cell activation. Sci. Signal..

[4] Zhu J, Yamane H, Paul WE (2010). Differentiation of effector CD4 T cell populations (*). Annu. Rev. Immunol..

[5] Kaech SM, Wherry EJ, Ahmed R (2002). Effector and memory T-cell differentiation: implications for vaccine development. Nat. Rev. Immunol..

[6] Szabo SJ (2000). A novel transcription factor, T-bet, directs Th1 lineage commitment. Cell.

[7] O’Shea JJ, Paul WE (2010). Mechanisms underlying lineage commitment and plasticity of helper CD4+ T cells. Science.

[8] van Panhuys N, Klauschen F, Germain RN (2014). T-cell-receptor-dependent signal intensity dominantly controls CD4(+) T cell polarization in vivo. Immunity.

[9] Zheng W, Flavell RA (1997). The transcription factor GATA-3 is necessary and sufficient for Th2 cytokine gene expression in CD4 T cells. Cell.

[10] Hale JS, Frock RL, Mamman SA, Fink PJ, Kennedy BK (2010). Cell-extrinsic defective lymphocyte development in Lmna(-/-) mice. PLoS ONE.

[11] Swain SL, McKinstry KK, Strutt TM (2012). Expanding roles for CD4(+) T cells in immunity to viruses. Nat. Rev. Immunol..

[12] Munier CM (2016). The primary immune response to Vaccinia virus vaccination includes cells with a distinct cytotoxic effector CD4 T-cell phenotype. Vaccine.

[13] Sen D, Forrest L, Kepler TB, Parker I, Cahalan MD (2010). Selective and site-specific mobilization of dermal dendritic cells and Langerhans cells by Th1- and Th2-polarizing adjuvants. Proc. Natl. Acad. Sci. USA.

[14] Sokol CL, Barton GM, Farr AG, Medzhitov R (2008). A mechanism for the initiation of allergen-induced T helper type 2 responses. Nat. Immunol..

[15] Mougneau E, Bihl F, Glaichenhaus N (2011). Cell biology and immunology of Leishmania. Immunol. Rev..

[16] Sacks D, Noben-Trauth N (2002). The immunology of susceptibility and resistance to *Leishmania major* in mice. Nat. Rev. Immunol..

[17] Mota BE (2011). Adverse events post smallpox-vaccination: insights from tail scarification infection in mice with *Vaccinia virus*. PLoS ONE.

[18] Qui HZ (2011). CD134 plus CD137 dual costimulation induces Eomesodermin in CD4 T cells to program cytotoxic Th1 differentiation. J. Immunol..

[19] Juno JA (2017). Cytotoxic CD4 T cells-friend or foe during viral infection?. Front. Immunol..

[20] Iborra S (2011). H-ras and N-ras are dispensable for T-cell development and activation but critical for protective Th1 immunity. Blood.

[21] Rocha-Perugini V, Gonzalez-Granado JM (2014). Nuclear envelope lamin-A as a coordinator of T cell activation. Nucleus.

[22] Lee K (2010). Mammalian target of rapamycin protein complex 2 regulates differentiation of Th1 and Th2 cell subsets via distinct signaling pathways. Immunity.

[23] Lund E, Collas P (2013). Nuclear lamins: making contacts with promoters. Nucleus.

[24] Gonzalez JM, Navarro-Puche A, Casar B, Crespo P, Andres V (2008). Fast regulation of AP-1 activity through interaction of lamin A/C, ERK1/2, and c-Fos at the nuclear envelope. J. Cell Biol..

[25] Ivorra C (2006). A mechanism of AP-1 suppression through interaction of c-Fos with lamin A/C. Genes Dev..

[26] van Panhuys N (2016). TCR signal strength alters T-DC activation and interaction times and directs the outcome of differentiation. Front. Immunol..

[27] Constant SL, Bottomly K (1997). Induction of Th1 and Th2 CD4+ T cell responses: the alternative approaches. Annu. Rev. Immunol..

[28] Komai-Koma M (2016). Interleukin-33 promoting Th1 lymphocyte differentiation dependents on IL-12. Immunobiology.

[29] Reithinger R (2007). Cutaneous leishmaniasis. Lancet Infect. Dis..

[30] Belkaid Y (2002). CD8+ T cells are required for primary immunity in C57BL/6 mice following low-dose, intradermal challenge with *Leishmania major*. J. Immunol..

[31] Uzonna JE, Joyce KL, Scott P (2004). Low dose *Leishmania major* promotes a transient T helper cell type 2 response that is down-regulated by interferon gamma-producing CD8+T cells. J. Exp. Med..

[32] Novais FO (2013). Cytotoxic T cells mediate pathology and metastasis in cutaneous leishmaniasis. PLoS Pathog..

[33] Scott P, Novais FO (2016). Cutaneous leishmaniasis: immune responses in protection and pathogenesis. Nat. Rev. Immunol..

[34] Iborra S (2016). Leishmania uses mincle to target an inhibitory ITAM signaling pathway in dendritic cells that dampens adaptive immunity to infection. Immunity.

[35] Scott P, Natovitz P, Coffman RL, Pearce E, Sher A (1988). Immunoregulation of cutaneous leishmaniasis. T cell lines that transfer protective immunity or exacerbation belong to different T helper subsets and respond to distinct parasite antigens. J. Exp. Med..

[36] Sullivan T (1999). Loss of A-type lamin expression compromises nuclear envelope integrity leading to muscular dystrophy. J. Cell Biol..

[37] Kim Y, Zheng X, Zheng Y (2013). Proliferation and differentiation of mouse embryonic stem cells lacking all lamins. Cell Res..

[38] Iborra S (2016). Optimal generation of tissue-resident but not circulating memory T cells during viral infection requires crosspriming by DNGR-1+ dendritic cells. Immunity.

[39] Rocha-Perugini V (2017). CD9 regulates MHC-II trafficking in monocyte-derived dendritic cells. Mol. Cell. Biol..

[40] Melamed S (2007). Tail scarification with *Vaccinia virus* Lister as a model for evaluation of smallpox vaccine potency in mice. Vaccine.

[41] Iborra S (2005). Vaccination with the *Leishmania infantum* acidic ribosomal P0 protein plus CpG oligodeoxynucleotides induces protection against cutaneous leishmaniasis in C57BL/6 mice but does not prevent progressive disease in BALB/c mice. Infect. Immun..

