# Supplementary figures and images for: Lamin A/C augments Th1 differentiation and response against vaccinia virus and Leishmania major
Source: Cell Death Dis. 2018 Jan 8;9(1):9. doi: 10.1038/s41419-017-0007-6 (PMC5849043; doi:10.1038/s41419-017-0007-6)

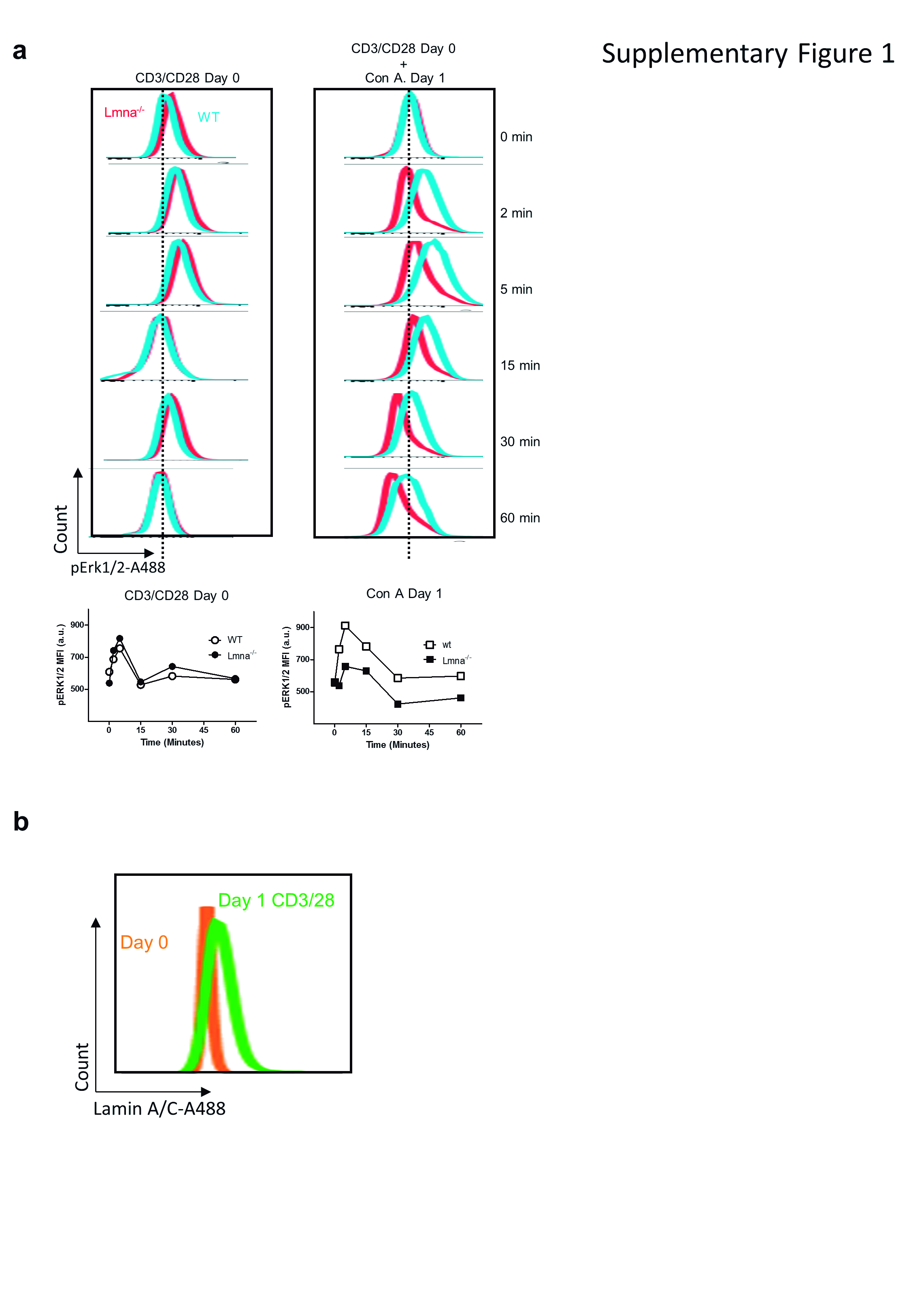

Supplement: Supplementary file 1 — Figure S1 [file 41419_2017_7_MOESM1_ESM.tif]

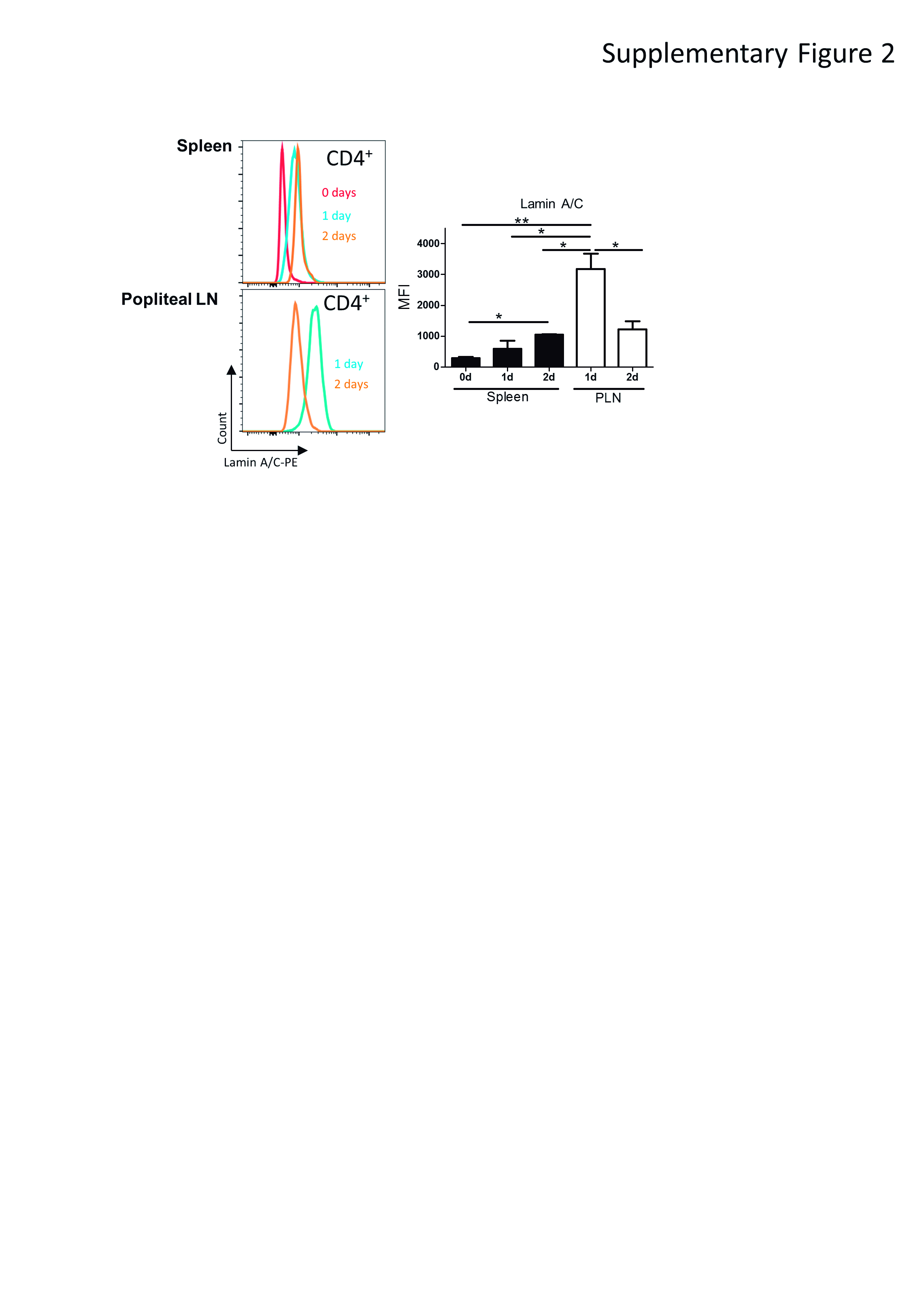

Supplement: Supplementary file 2 — Figure S2 [file 41419_2017_7_MOESM2_ESM.tif]

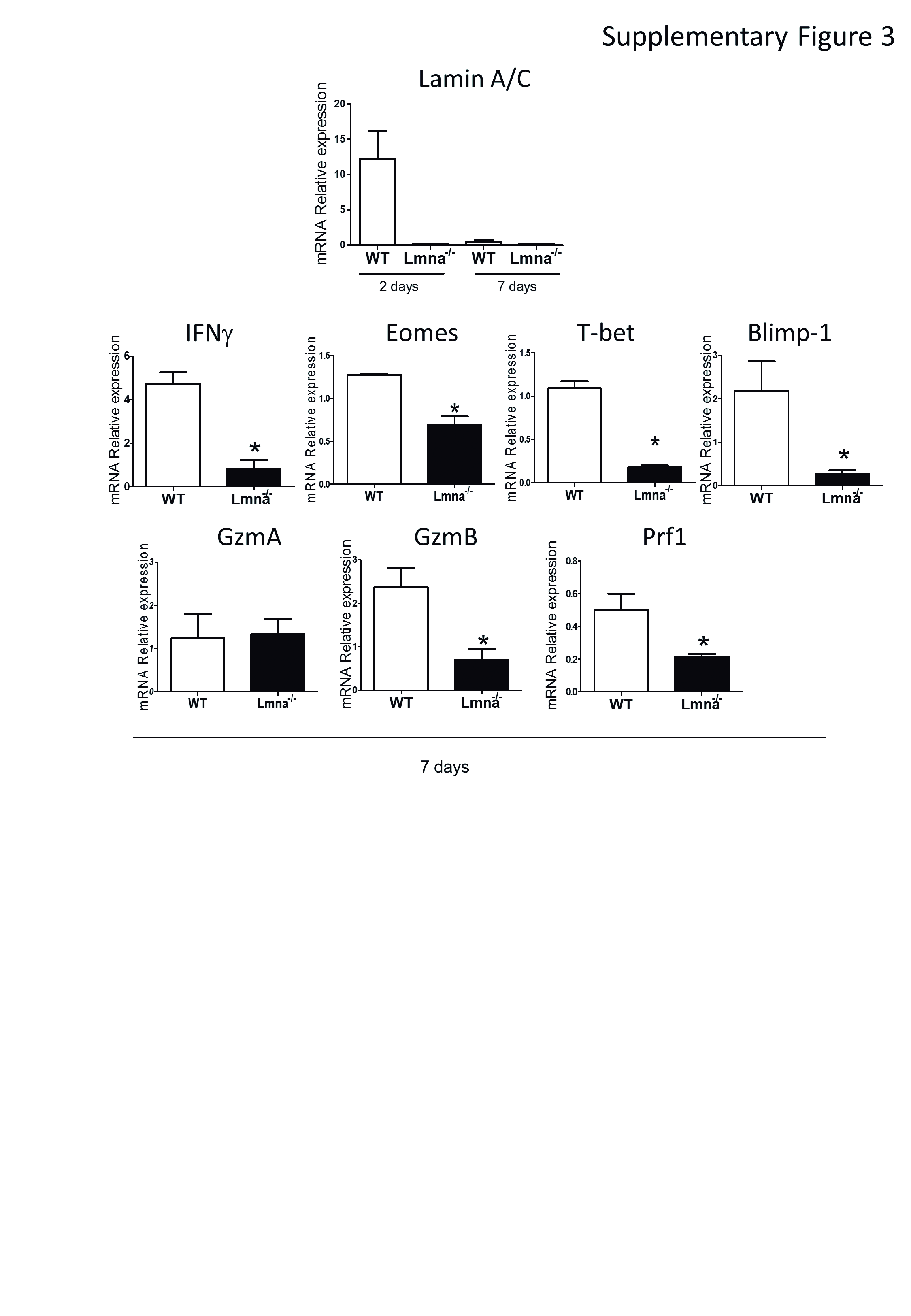

Supplement: Supplementary file 3 — Figure S3 [file 41419_2017_7_MOESM3_ESM.tif]

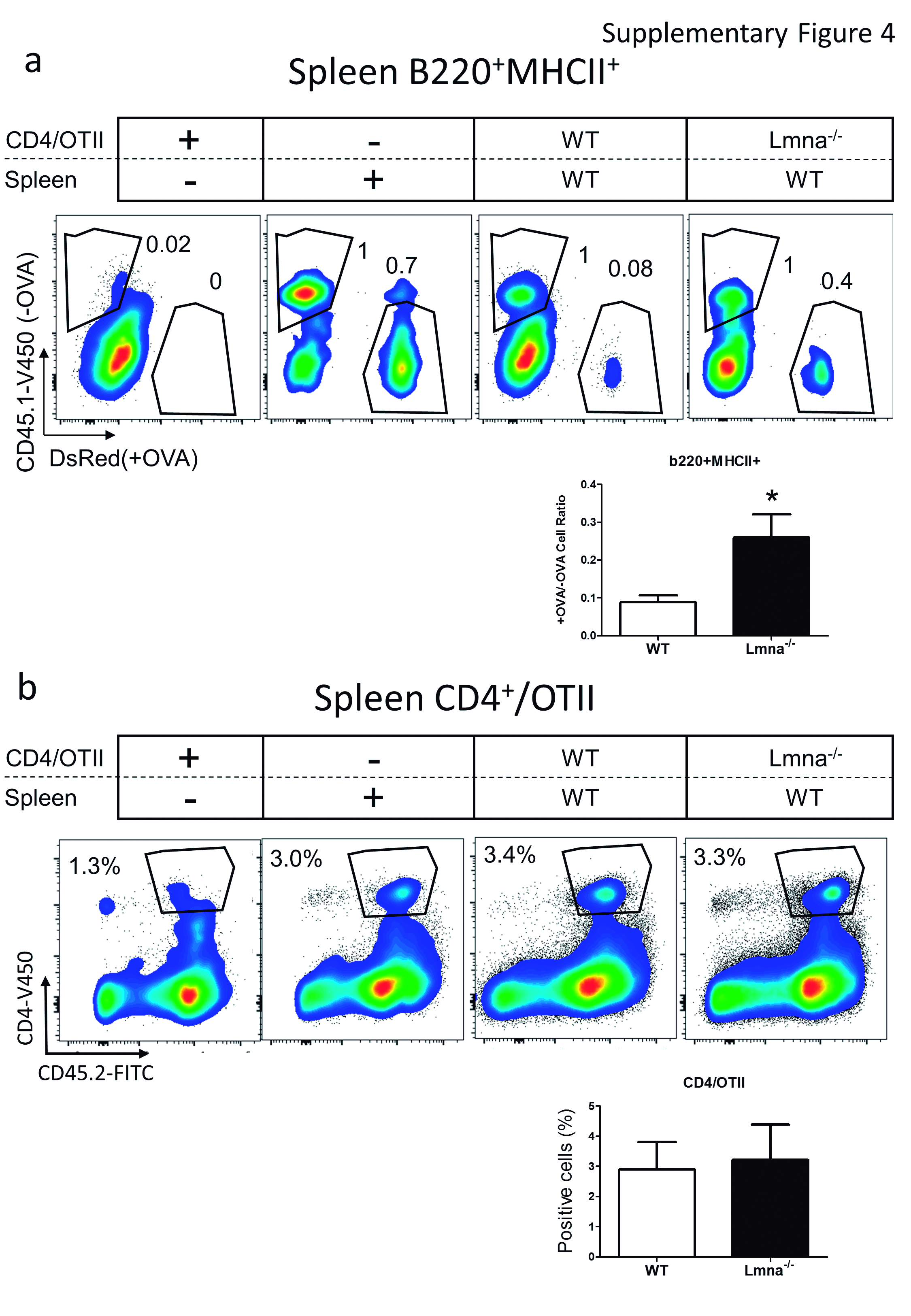

Supplement: Supplementary file 4 — Figure S4 [file 41419_2017_7_MOESM4_ESM.tif]
