# Supplementary material for: Lamin A/C augments Th1 differentiation and response against vaccinia virus and Leishmania major
Source: Cell Death Dis. 2018 Jan 8;9(1):9. doi: 10.1038/s41419-017-0007-6 (PMC5849043; doi:10.1038/s41419-017-0007-6)
Supplement: Supplementary file 5 — Supplementary Information [file 41419_2017_7_MOESM5_ESM.docx]

Supplementary Materials:

Figure S1. Naïve CD4 T-cells from *Lmna^-/-^* display normal early TCR-dependent signaling. **(a)** Intracellular levels of phosphorylated ERK1/2 was measured at short times after stimulation with anti-CD3/CD28 (left panels), and at short times after a second activation challenge with concanavalin A performed 1 day later (right panels). Data show a representative experiment (n = 1 pool of 3 mice). **(b)** Lamin A/C expression in unstimulated CD4^+^ T-cells (day 0) or in CD4^+^ T-cells that were stimulated with antibodies against CD3 and CD28 (1 day CD3/CD28).

**Figure S2.** **A-type lamins are expressed in CD4^+^ T-cells *in vivo* upon VACV infection. WT mice were inoculated or not with VACV intradermally in the footpad**. Plots and graphs show the amount of A-type lamins 1 or 2 days later (n=2 mice). Data are means ± SEM analyzed by One-way ANOVA with Bonferroni’s post-hoc multiple comparison test. *, P<0.05; **, P<0.01; ***, P<0.001

**Figure S3.** **Lamin A/C regulates IFNγ, T-bet, Eomes, Blimp-1, GzmB and Prf1mRNA expression in Th1 differentiating cells**. CD4^+^CD25^-^ T-cells were isolated from spleens of WT and *Lmna^-/-^* mice and activated with anti-CD3/CD28 antibodies for 2 days. Some cells remained in culture with Th1 polarizing cytokines for additional 5 days. Graphs show the mRNA levels of the indicated genes quantified by RT-qPCR at 2 or 7 days. Data are means ± SEM of at least two independent experiments (n = pools of 3 mice) analyzed by Student’s *t*-test. *, P<0.05; **, P<0.01; ***, P<0.001.

**Figure S4**. **Lamin A/C enhances the cytotoxic capacity of CD4^+^ T-cells *in vivo*.** *Rag1^-/-^* mice were inoculated intravenously with either naïve CD45.2/WT or CD45.2/*Lmna^-/-^* CD4^+^/OTII T-cells, and infected with VACV-OVA intraperitoneally. After 5 days, splenocytes from CD45.2/WT/dsRED and CD45.1/WT mice were isolated and loaded or not with OVA peptide, respectively. A mix approximately 1:1 CD45.2/WT/dsRED+OVA and CD45.1/WT-OVA splenocytes were inoculated intravenously in the recipient *Rag1^-/-^* mice, which were previously inoculated and infected. After 16 hours, spleens were collected and analyzed by flow cytometry for the : **(a)** the killing capacity of CD4/OTII cells of each genotype, by calculating the ratio between CD45.2/WT/dsRED/+OVA and CD45.1/WT/-OVA cells; and **(b)** the percentage of CD4/OTII T-cells. Mice adoptively transferred with CD4/OTII T-cells only, and mice transferred with splenocytes in the absence of CD4/OTII T-cells were used as controls (n=8 WT and 8 *Lmna^-/-^* mice from two independent experiments). Data are means ± SEM analyzed by unpaired Student’s t-test. *P<0.05.
